# CRISPR-directed exon skipping; friend or foe (opportunity or warning sign)?

**DOI:** 10.1016/j.omtn.2025.102539

**Published:** 2025-04-29

**Authors:** Kelly H. Banas, Eric B. Kmiec

**Affiliations:** 1 Gene Editing Institute, ChristianaCare Health System, 550 S. College Avenue, Newark, DE 19713, USA

## Background and context

As CRISPR-directed gene editing advances toward clinical application, a heightened awareness of secondary reactions has begun to subsume the field, as undoubtedly it should. One of the benchmarks for any CRISPR-based therapy is a convincing argument that the therapeutic acts primarily at the target site with minimal leakage to secondary locations. Extensive genetic (re)engineering of Cas nuclease(s) and a myriad of single guide RNA (sgRNA) modifications aim to avoid genetic toxicity and enhance precision. Scientific groups continue to catalog alternative CRISPR systems or variants including base and prime editing, whose reaction components are less likely to act at unintended sites. While we do not diminish the importance of acquiring and evaluating data from off-target analytics, the process of profiling indel patterns has led to an interesting side bar discovery: unique sequences near or at the target site that appear to enable molecular rearrangements, known as exon skipping.^1^^,^^2^^,^^3^^,^^4^^,^^5^

These molecular gymnastics should not be unexpected because a double-strand break, directed by CRISPR, radiation, or other chemical agents, sets in motion a cascade of events, ultimately resulting in some degree of unfaithful repair.^6^^,^^7^ In evolutionary terms, elimination of exons might enable cells to respond to environmental change, an adaptation strategy that might increase the chance of survival.

## Exon skipping and gene editing

We believe CRISPR-directed exon skipping is an important unintended outcome of foundational gene editing, facilitated and perhaps controlled by the indel signature left behind by the action of CRISPR/Cas. Deciphering this code could lead to some degree of predictability of functional outcomes, a critical aspect to evaluating gene editing as a therapeutic platform. Specifically, we believe that the DNA signature left after cleavage and re-ligation, a so-called *Indel Code*, could reveal collateral effects of exon skipping on clinical outcomes.

Observations and reports surrounding the process of exon skipping are not new, and some researchers have spent a career trying to harness this phenomenon in the march toward treatment of Duchenne’s muscular dystrophy and other afflictions. A regular stream of reports continues to highlight the desire for a solution, but most fall short of expectations. Recently, studies with CRISPR/Cas designed to eliminate targeted exons from the final transcript have been reported and may provide some hope for the sustainability of the approach.^8^ Is there an underlining code that can help improve the chances of success?

## Our journey through the minefield of exon skipping

We encountered exon skipping almost by accident during initial studies aimed at disrupting the NFE2L2 (NRF2) gene.^9^ Methodical characterization of clonal lung cancer cell populations isolated after disabling NRF2 revealed cells with variant transcripts, many with single or multiple exons missing. Since the objective of many CRISPR-based therapies is to disable the function of an intact gene (i.e., Casgevy), we recognized the significance of this CRISPR post-target effect. Thus, it became scientifically prudent to analyze the subgroups of edited populations generating different transcripts in detail. The critical part of this argument is that the totality of molecular outcomes could very well be masked in the short term, due to an overwhelming functional phenotypic response (an initial big bang!). Yet, unfortunate changes in population dynamics could gradually appear over time, influencing sustainability or even producing undesired outcomes through unknown environmental selectivity.

A consistent stream of evidence centered on the importance of exon skipping prompted us to revisit our own earlier observations. Specifically, we wondered whether an exon skipping event could account for the variation in the response to chemotherapy we had often observed within edited tumor cells. It had been well established that CRISPR-directed DNA breakage results in repaired, rejoined chromosomes that bear different genetic signatures at the junction point. If an exon skipping event had occurred in response to the breakage, a truncated or reconfigured mRNA could be produced and translated into a nonfunctional or partially functional protein. Could a random re-ligation process naturally create what we have termed the *Indel Code*?

**Figure 1:**
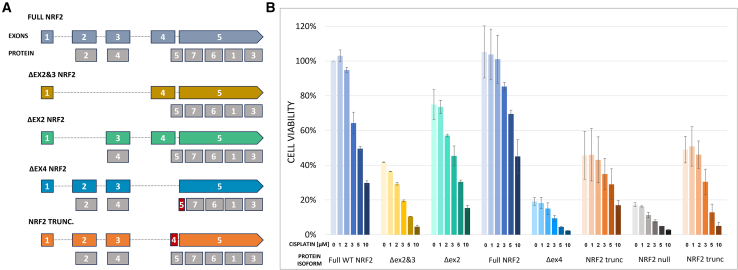
Cell viability of wild-type and NRF2-modified A549 clonal cell lines in response to cisplatin treatment (A) Schematic diagram of NRF2 splice variants present in each clonal cell line. (B) Cell viability was measured via bioreduction of MTS to a formazan product. Cells were treated with increasing concentrations of cisplatin for 72 h then evaluated for cell viability. The average cell viability normalized to untreated, full WT NRF2 is graphed and the error bars represent ± SEM.

We set out to reanalyze data generated and centered on understanding the genetic diversity of CRISPR/Cas activity.^9^ It was here that we confirmed exon skipping in NRF2. When comparing the loss of each exon, a variability in responsiveness to cisplatin exposure in each clonal population was observed (Figures 1A and 1B), for example, skipping of exon 2 and/or 3 results in the loss of the functional Neh2 and Neh4 domain, respectively. The loss of these domains impairs the functionality of the NRF2 protein, logically increasing chemosensitivity as compared to cells containing NRF2 with a full complement of exons. Cells lacking exon 2 also exhibited increased chemosensitivity, likely due to overall lower NRF2 expression. Exon 4 skipping displays heightened sensitivity to cisplatin even at low concentrations, reflecting the importance of this domain: a response like the clonal cell population devoid of any NRF2 (NRF2 null). So, it makes a difference how the *Indel Code* is written, and, in this case, the code reflects the allelic contribution of each subtype influencing protein functionality. These seemingly random positioned sequences have held a certain fascination for people involved in gene editing and exon skipping for some time.

While scientists have been trying to predict exon skipping, the process of chromosomal reclosure after a CRISPR-induced double-stranded break is unfaithful; therefore, electing a verifiable DNA sequence that initiates exon skipping in a gene editing experiment is likely to remain elusive. We think that the key now is to understand *how* the *Indel Code*, created by cleavage and closure, influences functional outcomes—something that should be quite relevant in developing CRISPR/Cas as a therapeutic tool. While reassured by our results, the exercise heightened our caution about recognizing this important phenomenon that undoubtedly occurs in many CRISPR-directed gene editing reactions.

## Friend of Foe?

*Foe:* exon skipping has now been shown to be a natural response to our collective attempts to edit the mammalian genome(s). As we have shown, the methodical expansion of sub-populations of targeted cells could reveal problematic bones that affect both the safety and the sustainability of therapeutic gene editing. No doubt, ESE/ESSs strewn throughout the genome might offer a portal to the code when altered by the action of CRISPR/Cas. It seems fair to proffer that the naturally occurring genetic rearrangements “post-editing” could generate a diversity of transcripts that needs to be considered more prominently per target gene. Using indel profiles, is it possible to elucidate the Indel Code that robustly predicts the functionality and/or the mutagenicity of genes targeted for disruption? Ultimately, it is a question of safety.

*Friend:* while the use of CRISPR-induced exon skipping has been historically centered around repairing a reading frame, its use is much broader. CRISPR is known to leave an uncontrollable footprint that can lead to indeterminate phenotypic outcomes. If the goal, however, is to functionally disable a protein by disrupting the gene, could one intentionally remove critical protein domains through the natural repair mechanism while exploiting controlled transcriptional regulation? We know we cannot control the genetic outcomes of CRISPR but by using CRISPR-induced exon skipping, could we control the phenotypic outcome? This is especially important when thinking about developing an *in vivo* oncology-based CRISPR therapy. Inherent heterogeneity of patients could play a role in the activity of CRISPR/Cas, thus dampening desired phenotypic outcomes; however, if we can intentionally disrupt the gene by targeting ESE/ESSs to induce strong exon skipping of critical protein domains, we can limit the heterogeneity of phenotypic outcomes, and that would be a remarkable use of a natural phenomenon.

## Acknowledgments

The authors recognize the important contributions made by members of the Kmiec research group at the Gene Editing Institute. This project was supported by grants from the 10.13039/100000057National Institute of General Medical Sciences (P20 GM103446 and P20 GM109021) and the Ammon Foundation. This content is solely the responsibility of the authors and does not necessarily represent the official views of the NIH.

## Declaration of interests

E.B.K. and K.H.B. are consultants to CorriXR Therapeutics.
